# Longitudinal changes of funduscopic optic disc size, color and cup-to-disc ratio in school children

**DOI:** 10.1186/s40942-024-00570-4

**Published:** 2024-07-25

**Authors:** Seiji Sameshima, Takehiro Yamashita, Hiroto Terasaki, Ryo Asaoka, Naoya Yoshihara, Naoko Kakiuchi, Taiji Sakamoto

**Affiliations:** 1 Department of Ophthalmology, Kagoshima University Graduate School of Medical and Dental Sciences, Kagoshima, Japan; 2 Department of Ophthalmology, Seirei Hamamatsu General Hospital, Shizuoka, Japan; 3 Seirei Christopher University, Shizuoka, Japan; 4 Nanovision Research Division, Research Institute of Electronics, Shizuoka University, Shizuoka, Japan; 5 The Graduate School for the Creation of New Photonics Industries, Shizuoka, Japan

**Keywords:** Optic disc color, Optic disc size, Cup-to-disc ratio, School myopia

## Abstract

**Background:**

To investigate the relationship between changes in the optic disc size and color, cup-to-disc (C/D) ratio, and axial elongation in schoolchildren.

**Methods:**

A prospective cohort study was performed in 75 right eyes of elementary school students for six years (from 8.5 to 14.5 years old). In the first and last year, all participants underwent optical axial length measurement and color fundus photography. The optic disc color was calculated by dividing the intensity of red by the sum of the intensity of red, green, and blue. The optic disc area was calculated by modifying the number of pixels according to Bennett’s formula. The C/D ratio was calculated by dividing the vertical cup diameter by vertical optic disc diameter. Wilcoxon signed rank test was used to compare these optic disc parameters and axial length in the first and last year.

**Results:**

Mean axial length in the last year (24.82 mm) was significantly longer than that in the first year (23.34 mm). Likewise, the mean optic disc size was significantly smaller in the last year (41,946 pixels) than that in the first year (46,144 pixels). The mean optic disc color in the last year (0.49) was significantly more reddish than that in the first year (0.46), while the mean C/D ratio in last year (0.50) was significantly smaller than that in first year (0.52).

**Conclusions:**

During the period from 8.5 years to 14.5 years of age, both the optic disc size and C/D ratio became smaller, while the color became more red.

## Background

The vertical cup-to-disc (C/D) ratio is a key indicator in glaucoma screening, alongside nerve fiber layer defects and disc hemorrhages [1–4]. The C/D ratio varies widely among individuals with normal eyes. While large discs themselves are not a concern, they may indicate the presence of glaucoma and are examined carefully. Conversely, small discs initially present with a low C/D ratio, potentially causing glaucomatous optic atrophy to be overlooked [5].

Individual variation in the C/D ratio in normal eyes is apparent from birth, with fundus photographs of premature infants showing ratios averaging between 0.12 and 0.32 with a standard deviation of 0.08 to 0.13 [6–8]. A longitudinal observational study on 184 eyes in children from 0 to 10 years of age revealed progressive optic cupping. In term children, the mean C/D ratio increased by 0.0075 per age-year, whereas in children born preterm, the rate nearly doubled at 0.0160 per age-year [9]. The C/D ratio in children has been reported in a number of cross-sectional studies: the vertical C/D ratio was reported to be 0.42 ± 0.15 in 1,309 eyes of 6-year-olds measured by OCT [10] and 0.39 ± 0.14 in 3,144 eyes of 7-year-olds [11], with a greater C/D ratio than at birth. From 0 years to 6 or 7 years of age, the C/D ratio become larger due to overall proportional enlargement of both the anterior and posterior segments [9–11].

However, during school age, the posterior eye is mainly elongated, especially in the equatorial region, and the proportions of the eye change from spherical to oval, resulting in progressive myopia [12, 13]. The mean and standard deviation of the C/D ratio by OCT in 135 normal eyes in individuals aged 4–18 years was 0.4 ± 0.2 at those aged 4–7 years, whereas the C/D ratio tended to be smaller at 0.3 ± 0.2 in those aged 8–12 years [14]. This phenomenon arises from the tilting of the optic nerve head in the context of oval eye enlargement, which causes the nasal nerve fibers to ascend over the optic nerve head, thereby reducing its size [15, 16].

Although the C/D ratio in schoolchildren has been reported in many cross-sectional studies [17–19], there are few reports of long-term observational studies. Cross-sectional studies encompass generational and individual differences, as well as changes with age. In contrast, long-term observational studies can directly investigate genuine age-related changes. Therefore, the purpose of this study was to clarify the changes in C/D ratio and optic nerve papilla size and coloration with ocular axis elongation in schoolchildren using a long-term observational study design.

## Methods

### Ethics statement

The methodologies employed adhered to the principles outlined in the Declaration of Helsinki and received approval from the Ethics Committee of Kagoshima University Hospital (Approval No.170,116(643)). Written informed assent and consent were obtained from all participants and their guardians. This study was registered with the University Hospital Medical Network Clinical Trials Registry (Registration No. UMIN000015239).

### Participants

This study was a longitudinal, prospective, observational investigation involving third-grade students aged 8 to 9 years during the initial examination [20, 21]. These students were enrolled at the Elementary School of the Faculty of Education, Kagoshima University. Out of the 144 third-grade students, written informed consent and assent were obtained from 122 (87.4%) students and their parents, respectively. The examinations took place from November 17 to December 18, 2014, during the first year (at the age of 8.5 years), and during the same period in 2020, the final year (at the age of 14.5 years). Only the right eyes were analyzed to avoid false confidence intervals and low P values. Exclusion criteria included: abnormal findings on fundus photography, systemic illnesses that affect the eyes, abnormal developmental history, previous trauma or ocular surgery, non-cooperation from child, truancy, transfer to or enrollment in another junior high school, instances where the peripheral fundus area lacked clarity, and eyes with unmeasurable fundus and ocular parameters. Fourteen eyes were excluded because of the difficulty in assessing the fundus and ocular parameters. Thirty-three students were excluded due to truancy, transfer, or enrollment in another junior high school. Ultimately, the current study included data from 75 people in the third grade (ages 8–9) in the elementary school and in the third grade (ages 14–15) in the junior high school. Color fundus photographs were captured using the 3D OCT-1 Maestro (Topcon, Tokyo, Japan), while axial length measurements were obtained using the OA-2000 Optical Biometer (Tomey, Nagoya, Japan).

### Measurements of optic disc size, optic disc color, and C/D ratio

**Fig. 1:**
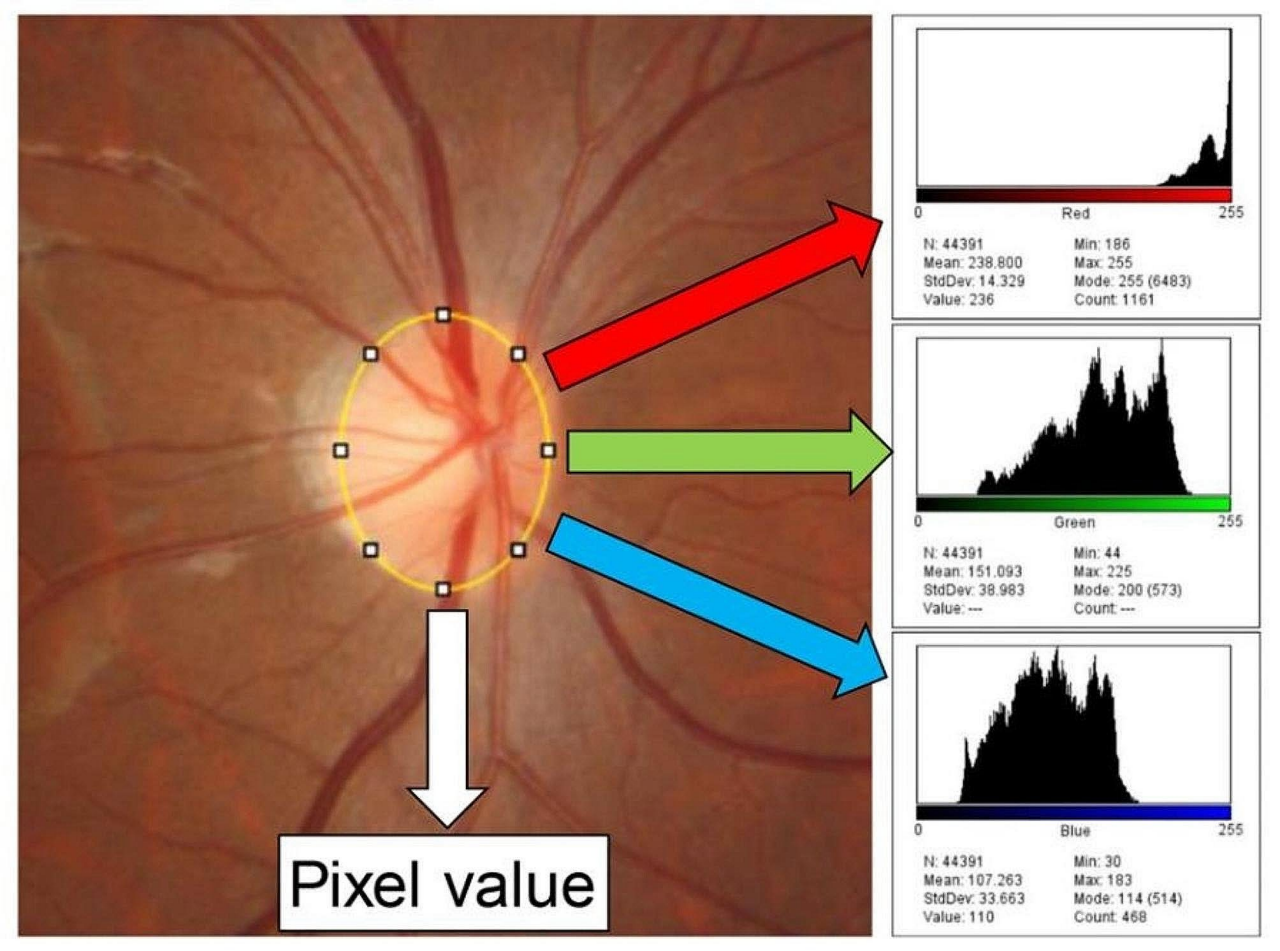
Measurements of optic disc size and color

Optic disc size was measured using ImageJ oval section tool software in first and last year fundus photographs (Fig. 1) [21, 22]. If the same angle of view is used, the image will be smaller as the fundus is farther away as the axial length elongation. When comparing fundus size in long-term studies of children, this magnification effect of the axial length was adjusted to reduce the size of the first-year photographs by Bennett’s formula (3.12 × 0.01306 × 100% / mm) [23, 24]. Vertical C/D ratio was measured by the vertical optic disc diameter from the vertical cup diameter.

The ImageJ software was utilized to identify and compute the mean red intensity (R), mean green intensity (G), and mean blue intensity (B) within the optic disc. Optic disc color was determined using the R, G, and B values according to the formula: R/(R + G + B) within the optic disc area [25]. Higher values indicate a redder optic disc color. All measurements were independently conducted by two masked examiners (TY, SS), and the average values were employed for analysis.

Pixel value is used for optic disc size. Magnification effect of the axial length was adjusted to reduce the size of the first-year photographs by Bennett’s formula. Optic disc color was calculated by dividing mean red intensity by sum of mean red, green, and blue intensity in optic disc area.

### Statistical analyses

All statistical analyses were performed with the SPSS statistics 21 for Windows (SPSS Inc., IBM, Somers, New York, USA). The inter-rater correlation coefficients of the optic disc parameters were calculated using a two-way mixed-effects model to measure absolute agreement. The axial length, optic disc size, optic disc color, and C/D ratio difference between 8.5 years and 14.5 years were analyzed using Wilcoxon signed rank test. Correlation among axial length, optic disc size, optic disc color, and C/D ratio at 8.5 years or 14.5 years was analyzed using Spearman’s correlation. Correlation among amount of change in axial length, optic disc size, optic disc color, and C/D ratio were also analyzed using Spearman’s correlation. *P* < 0.05 was considered to indicate statistical significance.

## Results

The participants included 37 boys and 38 girls. The inter-rater correlation coefficients of optic disc size, optic disc color, and C/D ratio at 8.5 years old were 0.965, 0.999, and 0.702, respectively (*P* < 0.001). These at 14.5 years were 0.957, 0.997, 0.684, respectively (*P* < 0.001). Ocular parameters for both groups are shown in Table 1. Over the six years, the axial length became significantly longer (*P* < 0.001), the optic disc area became significantly smaller (*P* < 0.001), the optic disc color became significantly redder (*P* < 0.001) and the vertical C/D ratio became significantly smaller (*P* = 0.004).

**Table 1:** Participants data

Ocular parameter	8.5 years old	14.5 years old	*P* value
Axial length (mm)	23.34 ± 0.92	24.82 ± 1.14	< 0.001
Optic disc size (pixels)	46,081 ± 8,997	41,946 ± 10,163	< 0.001
Optic disc color	0.46 ± 0.03	0.49 ± 0.03	< 0.001
Vertical cup-to-disc ratio	0.52 ± 0.09	0.50 ± 0.09	0.004

The relationship between optic disc parameters and axial length is shown in Table 2. At 8.5 years old, axial length was significantly and negatively associated with optic disc size (*r*=-0.45, *p* < 0.001), but not with optic disc color or C/D ratio. Optic disc size was negatively associated with optic disc color (*r*=-0.34, *p* = 0.003) and positively associated with C/D ratio (*r* = 0.39, *p* < 0.001), with a significant difference. At 14.5 years old, similar patterns were observed: axial length was significantly and negatively associated with optic disc size (*r*=-0.51, *p* < 0.001), but not with optic disc color or C/D ratio. Over the six years, axial length elongation showed no significant association with changes in optic disc size, color, or C/D ratio. However, changes in optic disc size were positively associated with changes in C/D ratio (*r* = 0.24, *p* = 0.04). Overall, optic disc color had no significant association with C/D ratio changes over the duration of the study period.

**Table 2:** Relationship between optic disc parameters and axial length

**8.5 years old**	Correlation coefficient (*p* value)			
	Optic disc size	Optic disc color	C/D ratio	Axial length
Optic disc size				
Optic disc color	-0.34 (0.003)			
C/D ratio	0.39 (< 0.001)	-0.31 (0.007)		
Axial length	-0.45 (< 0.001)	0.21 (0.08)	0.06 (0.64)	
**14.5 years old**	Correlation coefficient (p value)			
	Optic disc size	Optic disc color	C/D ratio	Axial length
Optic disc size				
Optic disc color	-0.38 (0.001)			
C/D ratio	0.44 (< 0.001)	-0.18 (0.13)		
Axial length	-0.51 (< 0.001)	0.18 (0.12)	-0.02 (0.89)	
**Changes**	Correlation coefficient (p value)			
	Optic disc size	Optic disc color	C/D ratio	Axial length
Optic disc size				
Optic disc color	-0.21 (0.08)			
C/D ratio	0.24 (0.04)	0.01 (0.96)		
Axial length	-0.10 (0.39)	0.04 (0.72)	-0.13 (0.28)	

C/D ratio: Cup-to-Disc ratio

**Fig. 2:**
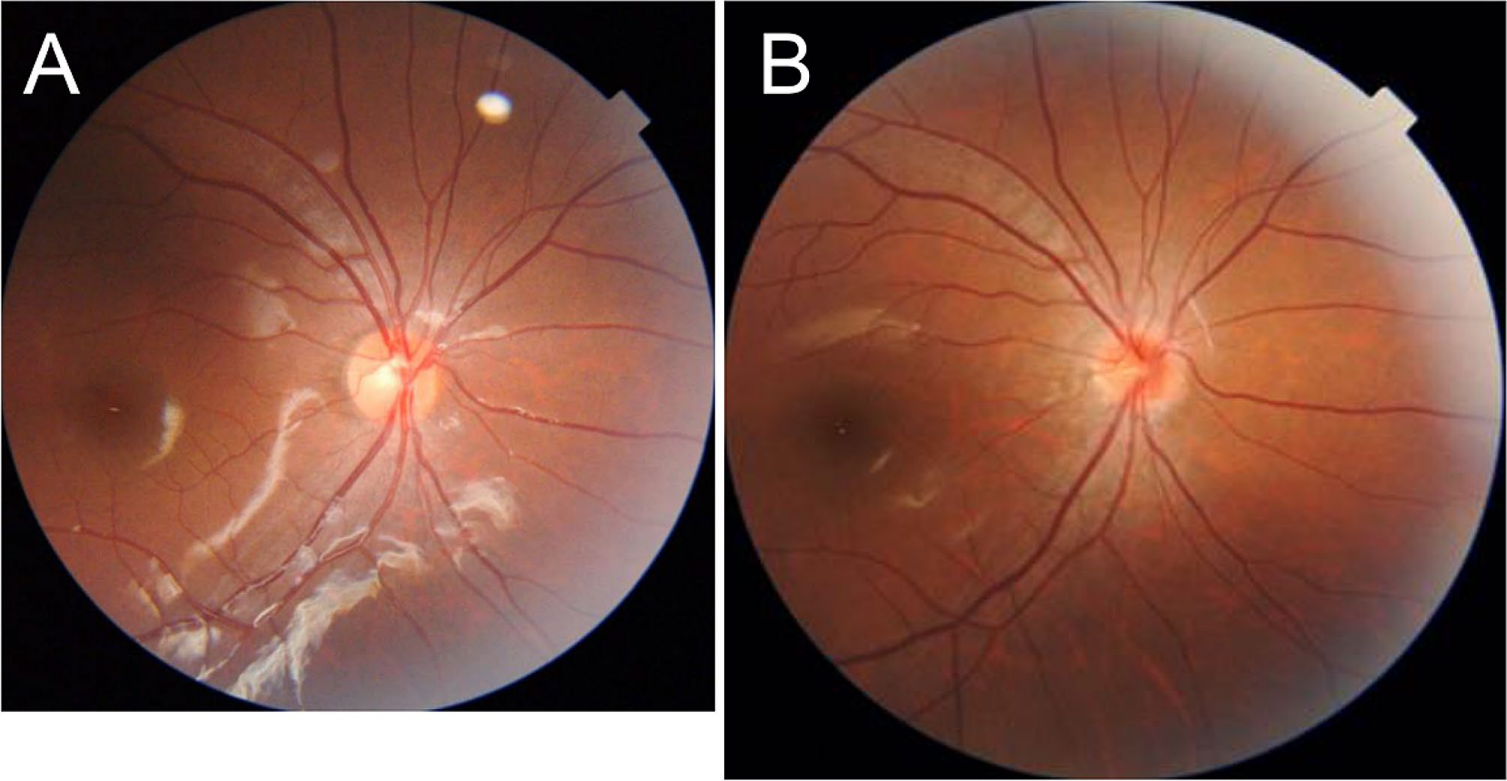
Representative case with optic disc size shrinkage, color reddening, and C/D ratio reduction. Redder optic disc color, smaller optic disc size, and smaller C/D ratio at last year (14.5 years old, **b**) compared with first year (8.5 years old, **a**). The image size in the first year was reduced using Bennet’s formula

A representative case with optic disc size shrinkage, color reddening, and C/D ratio reduction is shown in Fig. 2. Axial length was 22.51 and 24.51 mm, optic disc size was 33,962 and 23,383 pixels, optic disc color was 0.50 and 0.54, and C/D ratio was 0.40 and 0.31 at 8 (Fig. 2a) and 14 (Fig. 2b) years old, respectively.

## Discussion

The results showed that from the ages of 8.5 to 14.5 years, the axial length became significantly longer, the optic disc size became significantly smaller, the optic disc color became significantly redder, and the vertical C/D ratio became significantly smaller. During this period, as the optic disc became smaller, the density of nerve fibers passing through the scleral ring increased, causing the color to become redder, contrary to glaucoma cases, where the number of optic nerve fibers decreases, and the color gradually becomes paler.

During infancy, enlargement of the eyeballs results in both the anterior and posterior eye areas becoming larger and maintaining a spherical shape, typically without inducing myopia, except in special cases. During this phase, the C/D ratio increases because of the overall enlargement of the eye [8–10]. In school-aged children, the posterior aspect of the eye, especially areas other than the macula, elongate, leading to an oval shape both in front of and behind the eye. This elongation causes myopia to develop as the focus shifts forward from the retina [12, 13]. The optic nerve attached to nasal side of the eye extends backwards without much movement, hence the optic disc is pushed from the nasal side, causing the optic disc and C/D ratio to gradually become smaller.

In the present study, the optic disc size was converted from pixels to square millimeters using the built-in measurement apparatus of the fundus camera. The converted optic disc size was 2.77 ± 0.54 square millimeters at 8.5 years old and 2.51 ± 0.61 square millimeters at 14.5 years old. A previous cross-sectional study of Australian children showed that the optic disc size was 2.20 ± 0.39 square millimeters at 6 years old [10]. These findings suggest that the change in optic disc size showed a similar tendency as that of the C/D ratio. However, there may be ethnic and generational differences, thereby necessitating confirmatory long-term cohort studies on children from an earlier age.

Another significant finding concerns the color of the optic disc. While the pallor of the optic disc has long been considered a crucial diagnostic criterion for glaucoma, there are numerous confounding factors, making it challenging to rely solely on this indicator for diagnosis. Both at 8.5 and 14.5 years of age, longer axial length was significantly associated with smaller optic disc size and smaller optic disc area was significantly associated with the redder optic disc color and the smaller C/D ratio. When eyes with smaller optic disc size and C/D ratio develop glaucoma, the C/D ratio is underestimated and glaucoma is missed more often due to the inherently smaller C/D ratio, even when the cupping is enlarged. Furthermore, the present study newly found that the optic disc color is redder in eyes with smaller optic disc size. If the optic disc is originally redder, it is less likely to become pallor when the optic nerve decreases due to glaucoma, which may lead to glaucomatous optic neuropathy being underestimated or overlooked. In addition, if the small optic disc is combined with adult axial myopia, the optic nerve will gradually become smaller in adulthood. This combination, which may be a disc at risk (a small disc with no cupping), has been considered the main risk factor for the development of non-arteritic anterior ischemic optic neuropathy [26, 27].

Regarding the changes from 8.5 to 14.5 years of age, the C/D ratio changes were significantly positively associated with the optic disc size changes. This indicates that at age 8.5 years, the smaller optic disc size was significantly associated with the smaller C/D ratio, and a similar trend progressed by age 14.5 years, with a stronger correlation coefficient from 0.39 at age 8.5 to 0.44 at age 14.5. There was a similar tendency in the relationship between the optic disc color and the optic disc size, but the correlation between the changes did not reach the significance level, which may be due to either the optic disc color not changing as significantly as the C/D ratio or to the small number of cases.

Although there was a trend towards smaller optic disc size with longer axial length at both 8.5 and 14.5 years of age, optic disc color and C/D ratio were not significantly correlated with axial length. During infancy, as the ocular axis elongates, the C/D ratio tends to be larger due to proportional ocular enlargement [9–11]. However, in schoolchildren, greater elongation of the ocular axis in an oval shape [12, 13] can push the optic disc from the nasal side, causing it to become smaller. This phenomenon may lead to the absence of a correlation between axial length and optic disc color and C/D ratio.

Although axial length was negatively correlated with optic disc size in both 8.5 and 14.5 years of age, axial length elongation was not associated with optic disc size changes. This lack of association may be attributed to the relationship already establishing itself before 8.5 years of age, with changes not reaching a significant level during the study period. Further research is needed focusing on long-term studies or studies involving younger age groups.

We previously reported that an increase in axial length was significantly associated with slower visual field progression in the inferior hemifield in a longitudinal study of patients with adult primary open angle glaucoma [28]. These findings suggest that the association between changes in the optic disc and axial elongation might continue even in adults. In the near future, we plan to conduct a longitudinal study to investigate the optic disc changes, including not only the C/D ratio, but also the optic disc size, color, and ovality ratio in primary open angle glaucoma eyes.

The first limitation of this study is the small number of cases. A minimum of 50 cases is required for exploratory studies [29]. Although this study had a reasonable number of cases, a larger sample size could potentially yield significant correlations between the observed changes. Also, although Bennett’s formula is suitable for correction purposes, it may have led to under- or over-corrected optic disc sizes. It is known that changes in the inferior staphyloma and dome-shaped macula have already occurred in primary school children [20], and because the posterior part of the eye is distorted, accurate correction is difficult. Future research using MRI and other techniques is needed to confirm whether the optic disc has genuinely reduced in size. Another limitation of this study is the low inter-rater agreement of the C/D ratio. Assessing the C/D ratio is often challenging because the border of the optic disc cup is not clear, especially in crowded discs in children. Further confirmatory research is necessary. Due to higher prevalence of myopia among Asians [30–32], it is unclear whether the present study applies to other ethnic groups.

In conclusion, in this cohort study involving healthy children from 8.5 to 14.5 years of age, the axial length became significantly longer, the optic disc size became significantly smaller, the optic disc color became significantly redder, and the C/D ratio became significantly smaller. Longer axial length was associated with smaller optic disc size and smaller optic disc size was associated with smaller C/D ratio and redder optic disc color at 8.5 and 14.5 years of age. Changes in the shape and color of the optic disc during growth phase may provide insight into optic nerve-related diseases, including glaucoma and non-arteritic anterior ischemic optic neuropathy.

## Data Availability

The datasets used during this study are available from the corresponding author on reasonable request.

## Abbreviations

C/D: Cup-to-disc
